# Supplementary material for: Predicting visual function by interpreting a neuronal wiring diagram
Source: Nature. 2024 Oct 2;634(8032):113–23. doi: 10.1038/s41586-024-07953-5 (PMC11446822; doi:10.1038/s41586-024-07953-5)
Supplement: Supplementary file 6 — CRF and ERF predictions for individual TmY4 and TmY9 cells. Analogous to Supplementary Data 3, but for TmY target types. Shown are the top four monosynaptic pathways, the strongest pathway passing through each of the top ten intermediary types (ranking from Extended Data Fig. 7), and the trisynaptic pathway Tm1–TmY–Dm3–TmY (see the section entitled Prediction of spatial normalization). [file 41586_2024_7953_MOESM6_ESM.zip › DataS4/TmY9qΓèÑ/720575940615736223.pdf]

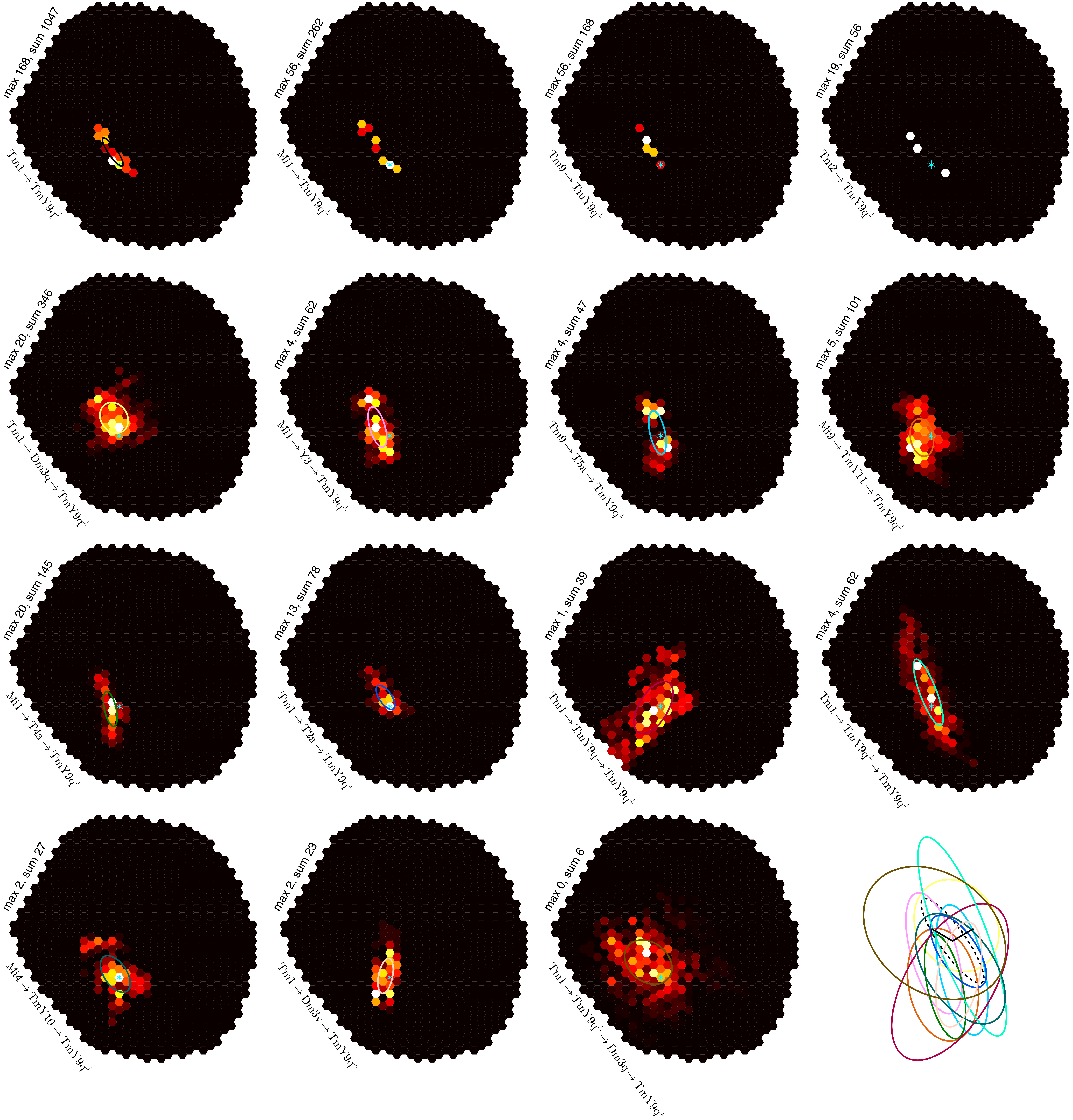

max 168, sum 1047
Tm1 → TmY9q⊥
max 56, sum 262
Mi1 → TmY9q⊥
max 56, sum 168
Tm9 → TmY9q⊥
max 19, sum 56
Tm2 → TmY9q⊥
max 20, sum 346
Tm1 → Dm3q → TmY9q⊥
max 4, sum 62
Mi1 → Y3 → TmY9q⊥
max 4, sum 47
Tm9 → T5a → TmY9q⊥
max 5, sum 101
Mi9 → TmY11 → TmY9q⊥
max 20, sum 145
Mi1 → T4a → TmY9q⊥
max 13, sum 78
Tm1 → T2a → TmY9q⊥
max 1, sum 39
Tm1 → TmY9q⊥ → TmY9q⊥
max 4, sum 62
Tm1 → TmY9q⊥ → TmY9q⊥
max 2, sum 27
Mi4 → TmY10 → TmY9q⊥
max 2, sum 23
Tm1 → Dm3v → TmY9q⊥
max 0, sum 6
Tm1 → TmY9q⊥ → Dm3q → TmY9q⊥
